# Evaluating Insects as Bioindicators of Heavy Metal Contamination and Accumulation near Industrial Area of Gujrat, Pakistan

**DOI:** 10.1155/2015/942751

**Published:** 2015-06-18

**Authors:** Iqra Azam, Sumera Afsheen, Ahmed Zia, Muqaddas Javed, Rashid Saeed, Muhammad Kaleem Sarwar, Bushra Munir

**Affiliations:** ^1^Department of Zoology, Institute of Life Sciences, University of Gujrat, Punjab 50700, Pakistan; ^2^National Insect Museum, National Agriculture Research Centre, Islamabad, Pakistan; ^3^Department of Statistics, University of Gujrat, Punjab 50700, Pakistan; ^4^Department of Environmental Sciences, Institute of Life Sciences, University of Gujrat, Punjab 50700, Pakistan; ^5^Department of Applied Chemistry and Biochemistry, Government College University of Faisalabad, Punjab 38000, Pakistan

## Abstract

To study the accumulation and contamination of heavy metals (i.e., Cd, Cr, Cu, Ni, and Zn) in soil, air, and water, few insect species were assayed as ecological indicators. Study area comes under industrial zone of district Gujrat of Punjab, Pakistan. Insects used as bioindicators included a libellulid dragonfly (*Crocothemis servilia*), an acridid grasshopper (*Oxya hyla hyla*), and a nymphalid butterfly (*Danaus chrysippus*) near industrial zone of Gujrat. Accumulation of Cd was highest in insect species followed by Cu, Cr, Zn, and Ni at *p* < 0.05. Hierarchical cluster analysis (HACA) was carried out to study metal accumulation level in all insects. Correlation and regression analysis confirmed HACA observations and declared concentration of heavy metals above permissible limits. Metal concentrations in insects were significantly higher near industries and nallahs in Gujrat and relatively higher concentrations of metals were found in Orthoptera than Odonata and Lepidoptera. The total metal concentrations in insects were pointed significantly higher at sites S3 (Mid of HalsiNala), S9 (End of HalsiNala), and S1 (Start of HalsiNala), whereas lowest value was detected at site S6 (Kalra Khasa) located far from industrial area. HACA indicates that these insect groups are potential indicators of metal contamination and can be used in biomonitoring.

## 1. Introduction

Natural ecosystems all over the world have been adversely affected by human interventions [1]. Modern farming, industrialization, and increased vehicular use have led to high concentrations of heavy metals such as lead, nickel, chromium, cadmium, aluminum, mercury, and zinc in the environment [2]. These toxic heavy metals are regularly getting into air, water, and soil, thereby becoming part of natural biogeochemical cycle [3]. Insects have strong relationship with ecology and are popularly used as bioindicators since long time [4]. Acute and chronic effects of heavy metals on various insects are frequently reported in the form of growth inhibition, developmental abnormalities, reduced reproduction, and decreased hatchability [5].

Among aquatic insects, dragonflies are taken as being most sensitive to habitat disturbance [6]. Their presence in any water body confirms its synthetic pollution-free status [7]. Ecologically they are good indicator of the condition of terrestrial as well as aquatic ecosystems [8, 9]. Butterflies and grasshoppers also have ecological fidelity and are sensitive to environmental changes and quality. According to Chen et al. [10] these insects have been successfully used as bioindicators for environmental pollution and heavy metals contaminations near industrial states and even within urban areas.

The reasons for using these insect species as indicator are follows: (1) use of several different taxa of different habitat gives more robust results, (2) a quantitative indicator value needs to be associated with the bioindicators, (3) there is similarity between different landscape features, (4) there is comparison of community, (5) these taxa can be reliably identified, sampled, and quantified, and (6) more than one family surely indicate species richness of an order [11]. At landscape level, strong correlation is found among grasshoppers (herbivore) and butterflies (nectivore and herbivore). Furthermore for bioindication of ecological change we used aquatic insect dragonfly (Order Odonata). They are considered as best ecological indicator in water and riparian systems. They give a rapid and sensitive response to accumulation of heavy metals [12].

Gujrat is an important industrial area of Punjab province of Pakistan. It is business bay for ceramics, fan, furniture, and leather industry. Qadir et al. [13] reported more than 1000 cottage-level to large-scale industrial units from this district. In recent past structure, ground water, and landscape of Gujrat have been reported to be rapidly degraded due to industrialization, increased urbanization, and modern agricultural activities leading to injudicious use of pesticides and fertilizers [14]. Keeping in view environmental and health concerns due to increased heavy metal contamination, present study was designed to investigate level of heavy metal contamination in district Gujrat by using insects as bioindicators.

## 2. Materials and Methods

**Figure 1:**
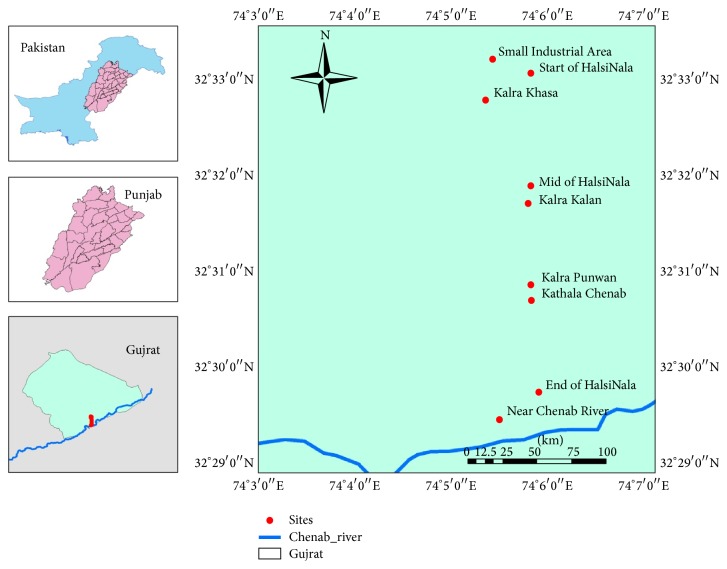
Map showing sampling sites (Arc GIS 9.3).

Survey to study heavy metal contamination was carried out during September 2013 to August 2014 in nine localities (Start of HalsiNala (S1), Kalra Punwan (S2), Mid of HalsiNala (S3), Small Industrial Area (S4), Kalra Kalan (S5), Kalra Khasa (S6), Kathala Chenab (S7), Near Chenab River (S8), and End of Nala (S9)) of Tehsil Gujrat (Figure 1). Details regarding coordinates and vegetation cover at each sampling site are shown in Table 1. Sites were selected at nearest possible distance to the functioning industrial units. Three insects groups were chosen to be used as bioindicators. It included a libellulid dragonfly (*Crocothemis servilia*), a nymphalid butterfly (*Danaus chrysippus*), and an acridid grasshopper (*Oxya hyla hyla*). These were collected from rice and wheat crops on quarterly basis for a period of one year. Three specimens of each group were recorded from each site so the total number of specimens (sample size) becomes twenty-seven for each species (Table 2). Soil and plant samples were also collected from same sites and stored in sterilized polythene bags until reaching laboratory. Represented specimens of each group were sent to National Insect Museum, NARC, Islamabad for taxonomic confirmation.

Gujrat is the most important industrial district of Punjab. Here the main sources of pollution in this region include smoke from chimneys, sewage water released into “HalsiNala”, and toxic waste dump sites. Contaminated water from “HalsiNala” is used for irrigation purposes in nearby fields that ultimately affect crops leading to heavy metal accumulation. Insects were collected from nine sites of industrial area (32°33′28.58′′N, 74°5′56.06′′E to 32°31′7.73′′N, 74°5′39.85′′E) for the assessment of heavy metals (Cu, Zn, Cd, Ni, and Cr).

Insects were mechanically dried in oven, weighed on microbalance, and digested in a mixture of supra pure grade nitric acid and perchloric acid mixed 4 : 1. Digests were analyzed for metal contents using atomic absorption spectrophotometer in an air-acetylene flame for zinc and copper and in a PU-93 090X graphite furnace for copper, chromium, cadmium, nickel and zinc.

Descriptive statistics, analysis of variance (one-way) ANOVA, bivariate correlation, and regression analysis were made on recorded data by testing significance differences at *p* < 0.05 using SPSS 16.0. Site dependent statistical differences between mean concentrations of heavy metals in each of the insect taxa were examined using descriptive statistics. Hierarchical cluster analysis (HACA) based on agglomerative statistics using Ward's Method was calculated for concentration of heavy metals at each of the sampling sites.

## 3. Results and Discussion

**Figure 2:**
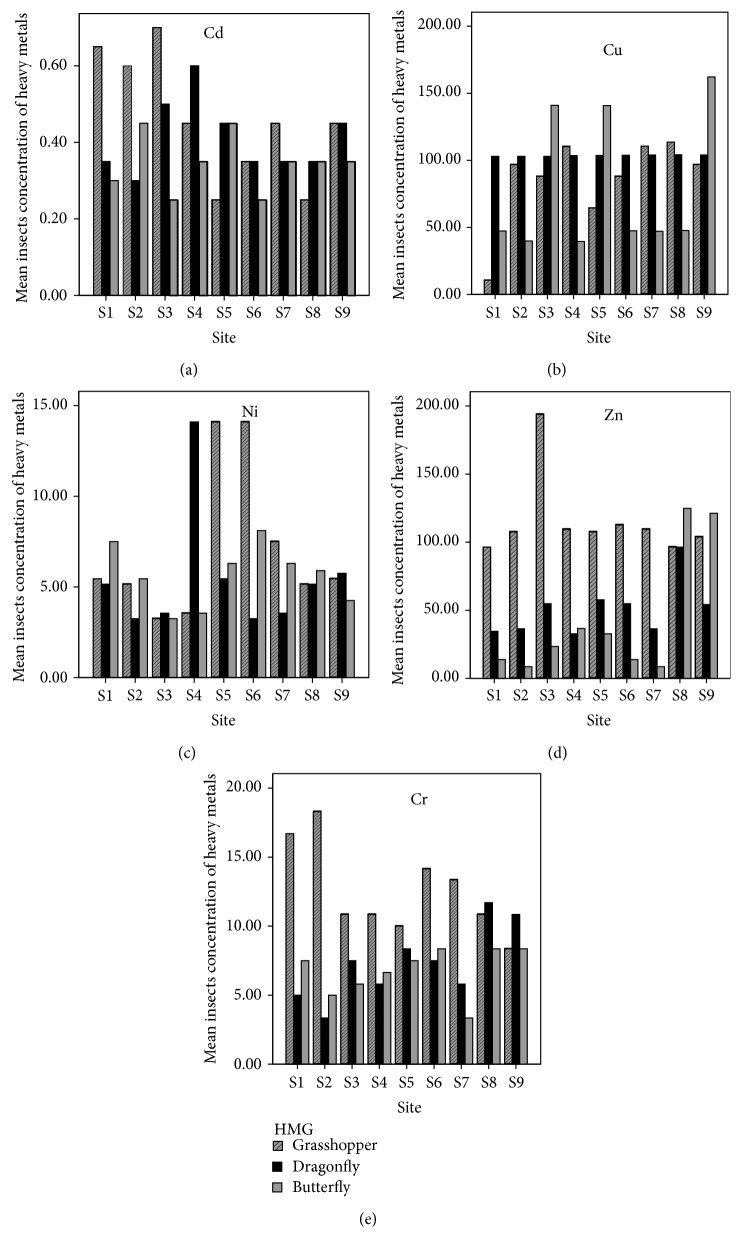
Mean values and standard deviations of metal concentrations in three insect taxa.

All studied sites generally showed remarkable species based variations in metal concentrations (Figure 2). Highest metal concentration was observed in the* Danaus chrysippus* from “End of Nala.” Significant site-based differences in levels of contamination were observed in the mean concentration of heavy metals at each site; however it varied up to threefold between examined species (Table 3). Lowest heavy metal concentration was seen in* Danaus chrysippus* specimens recorded from Kalra Punwan (S2). Apparent species dependent differences in metal pollution gradient were found in each examined species. All sampled soils were found to be slightly acidic, showing pH around neutrality (Table 4). Larger differences were however observed for organic matter contents and available phosphorus (Table 9). Descriptive statistics for soluble salts and other properties of water samples taken from nine sites is shown in Table 5.

Nonparametric test was also carried out for obtained data of this study. Two-independent-sample tests by Mann-Whitney test for two values and *K* independent tests by Kruskal-Wallis test and Jonckheere-Terpstra test were done to compare means of data. The distribution as shown in Table 7 indicates that season, site, crop, and HMG show insignificant results while metals show significant results at *p* < 0.05. It was thus likewise accepted that season, site, crop, and HMG have equal effect on accumulation of heavy metals in insect's body. On the other hand type of metal does not show equal effect and gave significant results at *p* < 0.05.

Variation within each parameter of soil and water showed their effect on each other (Table 7). EC, OM, AK, and S showed significant results in soil at *p* < 0.05, while AP displayed insignificant results and hence pH and AP showed equal effect. In water parameters all factors displayed significant results at 5% level of significance.

Correlations between metals were also studied and found predominantly positive in the case of Cd and Cr (Table 8). However it was observed to be positive as well as negative in case of Ni, Zn, and Cu. Zn concentrations were species-specific and correlation with Zn, Ni level in humus layer was statistically insignificant. Significant correlation was found between Cd, Zn, and Cr in* O. hyla hyla* Cd, Zn, and Cr in* C. servilia* (*p* < 0.05). But strongly positive relationship was observed between Zn and Cr in* D. chrysippus* and Zn, Cr, and Cd in* O. hyla hyla.* Similarly in* C. servilia* strong positive relationship was seen between Cr and Cu, Cr and Zn, Cd, and Ni. Body level of Cr, Cd correlated negatively with those of Ni and Cu in* O. hyla hyla* and* D. chrysippus* species except for* C. servilia.*

Table 11 explains the regression analysis of heavy metals group (*O. hyla hyla, C*.* servilia*, and* D. chrysippus*) using copper (Cu), chromium (Cr), and cadmium (Cd), as dependent for each insect group. By critically evaluating Table 11 it is concluded that the model of* O. hyla hyla* which contain copper (Cu) and chromium (Cr) as dependent is insignificant and the independent variables moderately explain the variation of dependent variables. Whereas the model of* O. hyla hyla* which contain cadmium as dependent variable and nickel (Ni), zinc (Zn), chromium (Cr), and copper (Cu) as independent is significant, independent variables can explain 88% variation of dependent variable and all *β* are significant. So overall we can conclude that the model which contains cadmium as dependent variable is suitable to explain metal toxicity in* O. hyla hyla*.

The models of* C*.* servilia* which contain copper (Cu) and chromium (Cr) as dependent are insignificant and the independent variables moderately explain the variation of dependent variables, whereas the model of* C*.* servilia* which contain cadmium (Cd) as dependent variable and nickel (Ni), zinc (Zn), chromium (Cr), and copper (Cu) as independent is significant. Independent variables can explain 88% variation of dependent variable and all *β* are significant. So overall we can conclude that the model which contains cadmium as dependent variable is suitable to explain the* C*.* servilia* metal toxicity.

In regression model for third group (*D. chrysippus*) which contain copper (Cu) and chromium (Cr) and cadmium (Cd) as dependent, all are insignificant. By critically observing values of all *β*, we conclude that overall model is weak. Hence copper (Cu) and chromium (Cr) and cadmium (Cd) cannot be used to represent the metal concentrations of all other metals. Our results of predictive model clearly demonstrated that these metals have different anthropogenic source and their concentrations are not explained relative to each other.

Hierarchical agglomerative cluster analysis (HACA) was also evaluated to assess concentration of heavy metals in urban soils and to check environmental quality; for each data set (three insect taxa) multivariate cluster analysis using SPSS 16.0 was applied to ascertain similarity between the sites. Metal concentration in three insect taxa (*O. hyla hyla, C. servilia, and D. chrysippus*) showed significant differences in accordance with land type at each site. Sites located within industrial area represented higher values of metals in insects than in sites far from them. Heavy metals presented major values in S5 (Small Industrial Area I) with industrial area (Table 6). Lowest value was detected in S6 (Kalra Khasa) located far from industrial area. Cluster analysis was applied to the values of metal concentration for three insect taxa at nine sites. S1 located at the start of Halsi Nala showed different pattern of concentration of metals. CA grouped sampling sites into clusters (called zones in this study) on the basis of similarities within a zone and dissimilarities between different zones. Result of CA helped in interpreting the data and indicating pattern of similar objects. Ward's method was used as a clustering technique to identify the zones in data and square Euclidean distance was used as a distance matrix.

**Figure 3:**
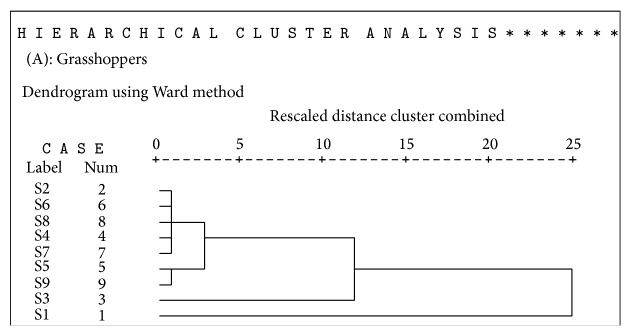
Dendrogram of the cluster analysis (using Ward's Method) applied for metal concentration in grasshoppers (*O. hyla hyla*) at nine sites.

**Figure 4:**
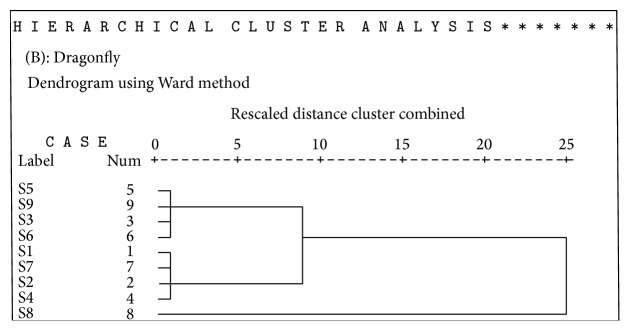
Dendrogram of the cluster analysis (using Ward's Method) applied for metal concentration in dragonfly (*C. servilia*) at nine sites.

**Figure 5:**
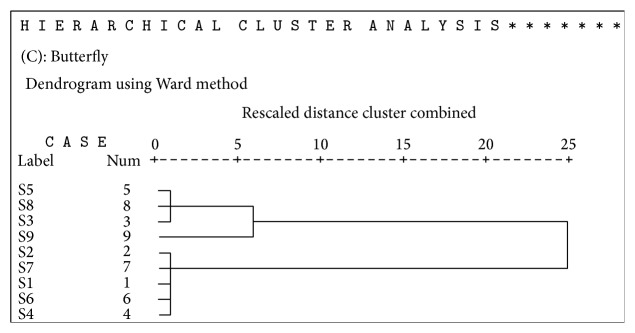
Dendrogram of the cluster analysis (using Ward's Method) applied for metal concentration in butterfly (*D. chrysippus*) at nine sites.

Three zones were identified using CA in each of the insect taxa (Figures 3, 4, and 5). Zone 1 consisted of highly polluted sites namely, 1 and 3 (Start of HalsiNala and Mid of HalsiNala). This zone was restricted to those places that were in close proximity to industrial stagnant waters and seasonal nallahs receiving large volumes of effluents and sewage waste throughout the year. Mid of HalsiNala had stagnant water in which pollutants from urban, industrial waste and agricultural runoff during monsoon are discharged. It is likely that seepage and infiltration of pollutants from nearby running HalsiNala affect the quality and purity of water and soil at this site. Generally level of measured heavy metals was low in this zone in comparison to sites 1, 3, and 9.

### 3.1. Discussion

Insects are typically the overwhelmingly dominant invertebrate faunal group and extensively used in biomonitoring and bioassessment programs throughout the world. Metals detected in our examined species are as a result of industrial effluents, agricultural runoff, vehicular smoke, domestic and sewage wastes, and use of fertilizers. As these metals are persistent and cannot be degraded by insect metabolism, hence these are accumulated at upper trophic level.

Analytical results obtained during present study are presented for all studied elements, season, insect taxa (species), and sites. As can be expected, insignificant differences were observed in metal concentration for all investigated sampled sites. In grasshoppers as a polyphagous and omnivorous insect, relatively higher contents of heavy metals were found at site “Start of HalsiNala” and “Mid of HalsiNala” that were nearer to the industrial area. As a predator, dragonflies (*C. servilia*) also had higher contents of heavy metals as it consumes many other insects of that area and thus accumulates high concentration of metals from aquatic environment compared to other two insect groups studied. Butterflies (*D. chrysippus*) being nectivorous comparatively showed less concentration of heavy metals as compared to other insect taxa studied. These results are in accordance with the observations of Li et al. [15] who point out that some metals contamination like Cd and Ni decreases with increased tropic levels.

In the current study all the three insect taxa clearly show site and species dependent metal accumulation patterns for Cd, Ni, Cr, Zn, and Cu. Significantly elevated concentrations were found for Cd, Cr, and Cu in “Mid of HalsiNala” > “End of Nala” > “End of Nala” and it thus confirms the expectation that animal body's burden reflects site pollution (*p* < 0.05). Less contrasting differences were found for Ni, while Zn ranges were not significantly different between all sites except at site 3. It might be assumed that in insect metabolism there are physiological mechanisms, which aid in regulations of metal ions concentrations and prevent toxic levels.

For each group of insect species, site and species dependent differences are recorded quite high which can be used as bioindicator of heavy metal stress at particular site and in particular species. Sampling localities found in vicinity of urban areas are suffering with heavy load of metal stress which ultimately results in higher concentrations of Cd, Ni, Cr, Zn, and Cu as compared to less polluted domains side by. For* O. viridulus* 59.39 mg/L of body loads of heavy metals is recorded which enable us to conclude that grasshoppers are good potential metal accumulators.

Taking into account that grasshoppers and butterflies are herbivorous insects and crop type is dependent on soil and water of specific sites, soil and water samples were taken for analysis from same sample sites. An analysis of correlation coefficients (linear, *r* ^2^) between metal concentrations and insects, and between soil and water parameters revealed significant (*p* < 0.05) relationship (Table 10). Our study found positive correlation between Cd, Cu, and Cr for each of the examined species, but not between Zn and Ni although Zn can be replaced by Cd.

Urban expansion and further development of industries, tanneries in the city would most likely enhance pollution. One of the major concerns is untreated industrial and municipal sewage pollution which poses major threat to the River Chenab, which is regarded as a pristine aquatic resource of Punjab province. Similarly lives of flora, fauna, and above all humans are at risk due to these untreated and regularly increasing industrial and urban wastes.

## 4. Conclusions

The present study highlights the extent of threat to insect and human lives in industrial area of Gujrat as a result of increasing metal concentration of Cu, Cr, Cd, Zn, and Ni. It also brings forward scope of different insects to be used as tool to study environment quality and conditions. The study thus advocates a need for proper measures to be taken to lessen increasing environmental pollution (soil, air, and water) by strictly implementing pollution control laws and enforcing proper disposal of industrial effluents in industrial zones.

## Figures and Tables

**Table 1:** Geographical location and vegetation type of sampling sites.

Sites selected for sampling	Site 1 Start of HalsiNala	Site 2 Kalra Punwan	Site 3 Mid of HalsiNala	Site 4 Small Industrial Area	Site 5 Kalra Kalan	Site 6 Kalra Khasa	Site 7 Kathala Chenab	Site 8 near the Chenab River	Site 9 End of Nala
Latitude	32°33′28.58′′N	32°32′53.64′′N	32°33′9.02′′N	32°33′58.70N	32°32′46.73′′N	32°32′44.98′′N	32°31′15.07′′N	32°29′25.7712′′N	32°31′7.73′′N
Longitude	74°5′56.06′′E	74°5′52.18′′E	74°5′21.57′′E	74°5′24.80′′E	74°5′2.50′′E	74°5′38.43′′E	74°6′5.53′′E	74°5′30.1992′′E	74°5′39.85′′E
Vegetation	Rice	Rice	Rice	Rice	Wheat	Wheat	Wheat	Wheat	Rice

**Table 2:** Sampling time along with temperature, humidity, and types of insect recorded.

	Season											
	Active season						Moderately active season					
Month (2013-14)	May 2014	June 2014	July 2014	Aug 2014	Sept 2013	Oct 2013	Nov 2013	Dec 2013	Jan 2013	Feb 2013	Mar 2013	April 2013

Type of crops (vegetation)	Rice	Rice	Rice	Rice	Rice	Rice	Rice	Wheat	Wheat	Wheat	Wheat	Wheat

Temperature (Celsius)	33°C	45°C	40°C	32°C	31°C	28°C	25°C	20°C	21°C	25°C	26°C	28°C

Dew point temperature TD (Celsius)	21°C	32°C	28°C	20°C	19°C	17°C	14°C	9°C	10°C	14°C	15°C	17°C

Relative humidity (*f*) percent	49.26%	49.02%	50.84%	49.03%	48.80%	51.23%	50.53%	49.22%	49.55%	50.53%	50.77%	51.23%

Types of insects collected	Butterfly	Butterfly grasshopper	Butterfly grasshopper	Grasshopper	Grasshopper dragonfly	Grasshopper dragonfly	Grasshopper dragonfly	Butterfly	Butterfly	Butterfly	Butterfly	Butterfly grasshoppers

**Table 3:** Mean concentrations of metals (mg/L) ± S.D. recorded in three insect taxa.

Site	Number	*O. hyla hyla *	*C. servilia *	*D. chrysippus *
Start of HalsiNala	5	25.96 ± 39.79	29.61 ± 43.21	15.20 ± 18.50
Kalra Punwan	5	45.74 ± 52.00	29.20 ± 43.80	11.80 ± 15.90
Mid of HalsiNala	5	59.39 ± 83.00	33.88 ± 44.57	34.70 ± 60.07
Small Industrial Area	5	46.98 ± 57.60	31.33 ± 42.14	17.33 ± 19.07
Kalra Kalan	5	39.30 ± 45.50	35.12 ± 44.74	37.55 ± 59.05
Kalra Khasa	5	45.92 ± 50.87	33.95 ± 44.97	15.50 ± 18.45
Kathala Chenab	5	48.27 ± 56.56	30.01 ± 43.81	13.13 ± 19.26
Near the Chenab River	5	45.25 ± 55.01	43.54 ± 51.99	37.41 ± 52.32
End of Nala	5	43.04 ± 52.55	35.05 ± 44.05	59.20 ± 76.60

**Table 4:** Descriptive statistics for pH, EC, OM, and other properties in urban soil of nine selected sites.

Soil parameters	*N*	Minimum	Maximum	Mean	Std. Deviation	Skewness	
						Statistic	Std. error
pH	9	4.30	7.60	7.0444	1.03816	−2.901	.717
Electrical conductivity m·mohs/cm	9	2.20	11.32	3.8556	2.86815	2.733	.717
Organic matter %age	9	.19	.76	.4344	.21858	.185	.717
Available Phosphorous (ppm)	9	3.00	11.00	6.3333	3.08221	.256	.717
Available potash (ppm)	9	60.00	220.00	1.2833*E*2	61.29029	.228	.717
Saturation % age	9	38.00	70.00	54.8889	14.32170	−.275	.717

**Table 5:** Descriptive statistics for soluble salts and other properties in water of nine selected sites.

Water parameters	*N*	Minimum	Maximum	Mean	Std. Deviation	Skewness	
						Statistic	Std. error
Soluble salts Tss (ppm)	9	505.00	1024.00	8.6289*E*2	147.47241	−2.017	.717
Ca^+ +^ Mg^+^	9	5.00	8.00	6.3333	1.03078	.905	.717
Na^+^	9	.05	4.67	2.1967	1.25510	.286	.717
HCO_3_^−^	9	4.50	8.50	6.6667	1.39194	−.263	.717
Cl^−^	9	.50	2.00	1.2778	.61802	.092	.717
Sodium absorption ratio (SAR)	9	.05	2.81	1.3800	.86757	.206	.717

**Table 6:** Test statistics for equal effect of each factor on heavy metal accumulation.

Group	Test	Statistics	Significance
Season	Mann-Whitney test	5522.00	0.823^*∗∗*^

Site	Kruskal-Wallis test	2.7	0.952^*∗∗*^
	Jonckheere-Terpstra test	12053.500	0.152

Crop	Mann-Whitney test	5522	0.823^*∗∗*^

HMG	Kruskal-Wallis test	3.225	0.521^*∗∗*^
	Jonckheere-Terpstra test	10216.500	0.868^*∗∗*^

Metal	Kruskal-Wallis test	164.050	0.000^*∗*^
	Jonckheere-Terpstra test	4539.00	0.000^*∗*^

^*∗*^Significant at 5%.

^*∗∗*^Significant at 1%.

**Table 7:** Variations observed within group in soil parameters.

Group 1	Group 2	Statistics	Significance
Soil parameters			
	EC	9.0	0.004^*∗*^
	OM	0.00	0.000^*∗*^
pH	AP	38.0	0.863
	AK	0.00	0.000^*∗*^
	S	0.00	0.000^*∗*^

Water parameters			
	Ca^+^	.000	0.000
	Na^+^	.000	0.000
SS (ppm)	HCO_3_^−^	.000	0.000
	Cl^−^	.000	0.000
	SAR	.000	0.000

^*∗*^Significant at *p* < 0.05.

**Table 8:** Correlations for each of the insect taxa.

Metals	*Oxya hyla hyla *					*Crocothemis servilia *					*Danaus chrysippus *				
	Cu	Ni	Zn	Cr	Cd	Cu	Ni	Zn	Cr	Cd	Cu	Ni	Zn	Cr	Cd
Cu	1					1	.03	.53	.70	−.06	1	−.42	.34	.24	.02
Ni	−.17	1					1	−.22	−.02	.77		1	−.28	.19	−.05
Zn	.09	−.25	1					1	.84	−.14			1	.57	.06
Cr	−.36	−.01	−.19	1					1	.14				1	−.19
Cd	−.34	−.60	.51	.45	1					1					1

**Table 9:** Correlation of soil parameters.

Soil parameters	pH	Electrical conductivity m·mohs/cm	Organic matter % age	Available phosphorous (ppm)	Available potash (ppm)	Saturation % age
pH	1					
Electrical conductivity m·mohs/cm	.120	1				
Organic matter % age	.414	.568	1			
Available phosphorous (ppm)	.401	.571	.999^*∗∗*^	1		
Available potash (ppm)	.413	.570	1.000^*∗∗*^	1.000^*∗∗*^	1	
Saturation % age	.416	.414	.856^*∗∗*^	.853^*∗∗*^	.853^*∗∗*^	1

^*∗*^Significant at *p* < 0.05.

^*∗∗*^Significant at *p* < 0.01.

**Table 10:** Correlation of water parameters.

Water parameters	Soluble salts Tss (ppm)	Ca^++^ Mg^+^	Na^+^	HCO_3_^−^	Cl^−^	Sodium absorption ratio (SAR)
Soluble salts Tss (ppm)	1	.597	.691^*∗*^	.719^*∗*^	.500	.578
Ca^++^ Mg^+^		1	−.116	.392	.523	−.254
Na^+^			1	.684^*∗*^	−.024	.850^*∗∗*^
HCO_3_^−^				1	−.170	.296
Cl^−^					1	.291
Sodium absorption ratio (SAR)						1

^*∗*^Significant at *p* < 0.05.

^*∗∗*^Significant at *p* < 0.01.

**Table 11:** Regression analysis for each of the insect taxa.

HMG group	Variables		Correlation	Regression coefficients					*R* square	Significance
	Dependent	Independent	*r*	${\tilde{\beta }}_{0}$	${\tilde{\beta }}_{1}$	${\tilde{\beta }}_{2}$	${\tilde{\beta }}_{3}$	${\tilde{\beta }}_{4}$	*R* ^2^	*p*
*O. hyla hyla *	Cu	Ni, Zn, Cr, and Cd	0.722	128.74	−6.2	1.684	3.370	−258.55	0.521	0.469
	Cr	Ni, Zn, Cu, and Cd	0.79	3.92	0.026	0.501	−0.08	26.25	0.63	0.30
	Cd	Ni, Zn, Cr, and Cu	0.939	.172	−0.002	−0.021	0.003	0.021	0.881	0.039

*C. servilia *	Cu	Ni, Zn, Cr, and Cd	0.722	128.74	−6.214	0.684	3.370	−258.55	0.521	0.469
	Cr	Ni, Zn, Cu, and Cd	0.797	3.92	0.026	0.501	−0.080	26.257	0.635	0.303
	Cd	Ni, Zn, Cr, and Cu	0.939	0.172	−0.002	−0.021	0.003	0.021	0.881	0.039

*D. chrysippus *	Cu	Ni, Zn, Cr, and Cd	0.547	75.446	−15.492	−0.17	10.88	52.27	0.299	0.785
	Cr	Ni, Zn, Cu, and Cd	0.751	4.27	0.008	0.482	0.024	−5.365	0.564	0.404
	Cd	Ni, Zn, Cr, and Cu	0.328	0.108	0.398	0.000	0.007	0.001	−0.019	0.968
